# Collaborative development of course feedback with students for Psyched Up. Put more in, get more out

**DOI:** 10.1192/bjo.2021.452

**Published:** 2021-06-18

**Authors:** Lois Zac-Williams, Alistair Cannon, Chloe Saunders, Shuo Zhang, Sae Kohara

**Affiliations:** 1 King's College London; 2 Core Trainee, South London and Maudsley NHS Foundation Trust

## Abstract

**Aims:**

To develop a responsive and sustainable template for long-term course evaluation for PsychED Up

To obtain rich, meaningful and specific feedback across multiple domains which can be translated into course improvements

To work collaboratively with students interested in medical education having previously participated in the course

To empower current students with the knowledge that their input is valuable

**Background:**

PsychED Up is an innovative extra-curricular course for 3rd year medical students at King's College London delivered by psychiatry trainees, senior students and actors. It is in its second year of running and focuses on the hidden curriculum in medicine, exploration of holistic care and communication skills at the mind-body interface. Input from people with lived experience is used to shape teaching.

**Method:**

Embedded evaluation in course development sessions thus engaging the entire faculty in evaluation processes at the start of the new term

- Decided evaluation focus
- Face-to-face discussions
- Survey for faculty to determine what specific feedback content would be most useful
- Finalised the questionnaire
- Collaborative design and refinement of questions, confirmed sub-sections and scope of questionnaire

**Result:**

Revised questionnaire:

- Included rationale at the start
- Tailored questions so faculty have more useful responses
- Greater quantity of prompted questions
- Specific questions for large group presentation, small group teaching, actors’ performances and students’ reflections
- Thoughtful combination of quantitative ratings and open-space questions
- Reduced time between course sessions and obtainment of feedback
- Quality and quantity of feedback
- High response rates: 32/30 (2 duplicates) mid-term, 29/30 end-of-term
- High-quality filling of open-space feedback allowed consolidation of themes to improve the course

**Conclusion:**

Co-designing the feedback form with previous students from the course and faculty brought focus to the questions. They were more specific and were organised into sub-sections for different domains. This led to responses that were relevant, enriched with depth and breadth and provided faculty with richer, more personalised responses. More detailed reflections in feedback were thought to be due to better student understanding of the rationale for questions, and knowledge that their input would help improve the course. We have set up a robust system for collecting long-term feedback for PsychED Up. We will continue to make iterative amendments, and supplement questionnaire feedback with focus groups.

